# A revised short version of the compassionate love scale for humanity (CLS-H-SF): evidence from item response theory analyses and validity testing

**DOI:** 10.1186/s40359-020-0386-9

**Published:** 2020-02-22

**Authors:** Francesca Chiesi, Chloe Lau, Donald H. Saklofske

**Affiliations:** 1 Department of Neuroscience, Psychology, Drug Area and Child’s Health (NEUROFARBA), Section of Psychology, University of Florence, Florence, Italy; 2 Department of Psychology, University of Western Ontario, London, Canada

**Keywords:** Compassionate love, Short form, Item response theory, Validity, Reliability

## Abstract

**Background:**

Compassionate love is defined as awareness and understanding of one’s suffering, connecting with the distress, and being emotionally and cognitively moved to alleviate suffering. The Compassionate Love Scale for Humanity (CLS-H) was developed to measure compassion towards strangers who need help and/or are vulnerable. The present study aimed to develop an abbreviated version of the CLS-H using item response theory to provide a precise and non-redundant compassion measure for use in research and practice.

**Methods:**

Undergraduate students (*N* = 790; 65.8% females) completed the CLS-H and other measures intended to establish external validity. Items for the short version were selected based on high amounts of information and taking into account the content coverage of the construct.

**Results:**

The shortened scale consisted of 9 items and performed well in measuring a large spectrum of the underlying construct with acceptable reliability. In terms of validity, the previously observed pattern of correlations was confirmed demonstrating positive associations between compassionate love and measures of self-esteem, positive affect, and life satisfaction, as well as negative associations with negative affect and anxiety.

**Conclusions:**

Using IRT, we obtained a brief, precise, and valid tool for assessing compassionate love.

## Background

Compassion is described as being aware of someone’s suffering and connecting with the distress (i.e., emotional resonance), understanding the universality of suffering in human experience, and being emotionally and cognitively moved to alleviate suffering. Additionally, compassion involves recognizing a commonality with the sufferer, acknowledging that we too could find ourselves in a similar position and also implicates being able to tolerate uncomfortable feelings (e.g., distress, anger, fear) that might be elicited by the person in distress [1]. Specifically, compassionate love has been defined as an “attitude toward other(s), either close others or strangers and including feelings, cognitions, and behaviors that are focused on caring, concern, tenderness, and an orientation toward supporting, helping, and understanding the other(s)” ([2], p., 630). In other words, as suggested by Kanov et al. [3], compassionate love consists of noticing (i.e., being aware of people’s adversity and discomfort), feeling (i.e., reacting emotionally to their suffering), and responding (i.e., wishing to act to alleviate people’s misery and distress).

Although there are some overlaps with associated concepts (e.g., empathy, kindness, and altruism), compassion and compassionate love have been defined as distinct constructs including thoughts, emotions, and behaviors directed to relatives, strangers, and humanity itself [1]. In contrast with empathy which may apply to a broader range of emotions like joy or anger, compassion is felt specifically in response to suffering. Moreover, empathy does not necessarily involve a behavioral activation and, as Sprecher and Fehr [2] argued, compassion is wider than empathy because it can be felt for humanity at large, rather than only for specific others. Similarly, altruism has a greater focus and impact on behaviors but altruistic acts may have a broader range of motivations than compassion. Compassion also includes elements beyond kindness, including recognizing and being touched by suffering; and likewise, kindness is different from compassion because kindness is not linked only to suffering [1].

International professional bodies in healthcare, education, and the justice system emphasize the importance of compassion with its link to pro-social behaviors and community action [1, 2, 4]. Additionally, compassion is believed to have a wide range of benefits, including better clinical outcomes, quality of care, satisfaction with services [5, 6], individual well-being, and positive predictors of mental health [7, 8]. Moreover, it can foster school teachers’ engagement and subjective well-being at work [9], and more generally affect the workplace climate [3, 9, 10]. Accordingly, empirical research is needed to identify the conditions that might promote and those that might impede compassionate love expression, to give additional insight into how compassionate love might be fostered in individuals and societies [3, 11], and to implement interventions that seek to enhance people’s ability to give and receive compassion [12, 13].

To facilitate adequate research on compassionate love, it is important to develop and analyze the utility of current methodologies for assessment. There are several scales that measure compassion and compassionate love [1, for a review]. Among them, one largely employed instrument is the Compassionate Love Scale for Humanity (CLS-H) [2] that assesses compassion towards strangers who need help and/or are vulnerable. The scale consists of 21 items related to feeling moved by other people’s suffering, understanding or imagining something about their condition as a fellow being, and being motivated to help them. Whereas Sprecher and Fehr [2] did not explicitly propose a factor structure prior to analysis, exploratory factor analyses yielded a single factor. Internal consistency was high and validity was supported by significant correlations in the expected directions with measures of empathy, helpfulness, volunteerism, prosocial behaviors, self-esteem, positive mood, and spiritual experiences [2, 14]. Nonetheless, the content validity of the scale has been questioned because items such as: “I feel happy when I see that others (strangers) are happy” and “I very much wish to be kind and good to fellow human beings” appear to tap empathy and kindness, respectively [1], while other items seem to assess altruism more so than compassionate love (e.g., “I would rather engage in actions that help others, even though they are strangers, than engage in actions that would help me.”).

Starting from this premise, the aim of the present study was to develop a shortened version of the CLS-H to obtain a brief scale that is more specifically focused on compassionate love and at the same time preserves adequate reliability and validity. The obtained brief scale should offer added value in both research and practice because an effective, concise, and more centered measure can be more appropriate for large, multivariate studies on compassionate love in which many tests and scales may need to be administered [15] or it might be employed in short surveys, for example, in healthcare or educational workplaces [3, 9, 10]. Furthermore, respondents may be less likely to experience boredom, loss of interest or perceive the questionnaires as time-demanding and redundant during administration of a short-form [16].

Previously, the Santa Clara brief compassion scale (SCBCS), a short 5-item version of the CLS-H, has been proposed [17]. However, this short scale was obtained adopting poor item selection criteria (i.e., items were chosen based on moderate means, high standard deviations, and high item-total correlations) and limited validity evidence was provided [1]. Thus, there is a need to adopt stronger criteria for item selection and to more rigorously investigate the psychometric characteristics of the shorter version in comparison to the long original form. Indeed, a reliable and valid scale is of great importance for researchers who need an abbreviated short-form of a measure without losing any of the scale’s efficiency and effectiveness.

To achieve this goal, we conducted an item response theory (IRT) analysis. To the best of our knowledge, this is the first study employing IRT for evaluating the measurement properties of the CLS-H and, in particular, for shortening the scale. IRT is a non-linear modeling technique that calculates the respondents’ probability of endorsing particular item response options taking into account the item and the respondent’s characteristics [18]. Parameter estimates are calculated for each item and the overall test’s ability to differentiate among respondents with different levels of the trait being measured. All these aspects together offer several advantages in both investigating measurement properties and shortening scales compared to classic test theory [19]. Indeed, IRT analysis makes it possible to identify weak or unnecessary items while taking into account the amount of information provided by them for each level of the measured trait through the *item information function* (IIF). Thus, on the basis of item information, we aimed to select items that convey the higher amount of information and assure adequate precision along the different levels of the trait. Additionally, in selecting the CLS-H items, we checked whether maximum precision exists for items while maintaining adequate construct coverage (i.e., if there was a correspondence between IRT selection criteria and the need to identifying items most closely related to the trait of interest).

Instead of providing a single value for reliability (e.g., Cronbach’s α), IRT provides the *test information function* (TIF), which evaluates the reliability of the test at different levels of the measured construct [18, 20]. More precisely, the TIF describes the information the test provides at each particular trait level (the higher the information, the smaller the error associated with the measurement). The TIF shows how reliable the test is along the whole range of trait scores (local reliability). Since the TIF is generated by aggregating the IIFs, longer tests will measure an examinee’s attribute with greater precision than shorter tests. Nonetheless, in the IRT framework, item selection can be done limiting the loss in information and ensuring that the shortened scale maintains an adequate amount of information along the trait continuum. Taken together, items that maximize measurement precision across the spectrum of the latent trait continuum can be selected for a concise and precise short-form.

Applying IRT, item parameters can be estimated based on responses to the items from both the original and the shortened forms and, once item parameters are estimated, comparable IRT scores on a given construct may be calculated for each respondent taking into account the two different patterns of items [19]. Thus, to test the correspondence of the original and abbreviated CLS-H versions individual IRT scores derived from the long and short forms were compared.

Finally, although IRT analyses helps in developing a short form and it allows confirming the accuracy of the obtained shortened scale in measuring the underlying construct, there is a need to provide validity evidence to confirm that the short form of a test meets the same standards of validity as the longer form. Indeed, an important goal in the development of any test’s short form should be to replicate the pattern of relationships established for the construct as measured by the long form of the test [15].

In sum, the goal of the current study was to shorten the CLS-H to provide researchers with an abbreviated form that maintains adequate validity and reliability and improve usefulness and applicability.

## Methods

### Participants and procedure

Undergraduate students (*N* = 790; 65.8% females) enrolled in a large university in Canada were recruited to participate in this study. Students’ ages ranged from 16 to 36 years (*M* = 18.93, *SD* = 1.86). Participants completed the online questionnaire to receive a credit towards a psychology course. Participants consented to participating in the study and were debriefed following the completion of the study. This study has been approved by the local research ethics board.

### Measures

#### Compassionate love scale

The CLS-H (stranger-humanity version) is a 21-item self-report measure that evaluates the degree to which an individual feels compassion or altruistic love towards strangers, selfless caring, and the motivation to help humanity (Cronbach’s α for the present sample = .94) [2]. Participants rated each item on a 7-point Likert-type scale (1 = *not at all true of me*, 7 = *very true of me*).

#### Playfulness

The Short Measure of Adult Playfulness (SMAP) [21] uses five items to assess adult playfulness, defined as a trait-like characteristic of reframing everyday situations in a pleasurable, intellectually stimulating, and joyful manner (Cronbach’s α in the present sample = .81). Respondents utilize a four-point scale (1 = *strongly disagree*, 4 = *strongly agree*) to evaluate each item. The initial validation study provided evidence for internal consistency, structural validity, and concurrent validity [21].

#### Self-esteem

The Rosenberg Self-Esteem Scale [22, 23] evaluates subjective emotional evaluation of an individual’s own worth based on one’s internal beliefs and self-concept (Cronbach’s α = .88). Participants rated each item on a four-point Likert scale (1 = *strongly disagree*, 4 = *strongly agree*). Past research has established that this scale exhibited strong internal consistency and test-retest reliability, as well as structural, convergent, and discriminant validity [22, 23].

#### Positive and negative affect

The Positive and Negative Affect Schedule (PANAS) [24] evaluates the extent to which an individual endorses positive and negative affective experiences. Participants rated 20 affective states on a four-point Likert scale (1 = *not at all*, 5 = *extremely*). In the present sample, Cronbach’s α were .87 and .90 for positive and negative affect, respectively.

#### Satisfaction with life

The Satisfaction with Life Scale (SWLS) [25, 26] evaluates the cognitive aspects of subjective well-being using a seven-point Likert scale (1 = *strongly disagree*, 7 = *strongly agree*). Previous literature demonstrated the SWLS shows strong internal consistency and external validity. In the present sample, internal consistency is good (α = .84).

### Data analysis

IRT analyses were performed using IRTPRO 4.0 [27]. IRT models use the original response data for estimating probabilities of responses as a function of the latent trait θ, which is defined as a continuous variable that conventionally has a mean of zero and *SD* of 1.0. This function describes the relation between the probability of endorsing a response given not only the respondent’s level of θ but also the item characteristics. IRT models provide item location and discrimination parameters that enable the evaluation of how well an item performs in measuring the underlying construct, the level of the construct targeted by the item, and the appropriateness of the response categories [28].

Samejima’s [29, 30] graded response model (GRM) was applied for the response format of polytomous data. A fundamental assumption underlying the GRM is the unidimensionality of the underlying construct. The decision for the application of a discrimination parameter was indicated based on variability in classical item–total correlations. The variability in item discrimination parameters within each factor would warrant inclusion in the IRT calibration. Hence, as a preliminary step, to assess the factor structure of the CLS-H, a parallel analysis was performed and an unrestricted factor analysis using a Robust Unweighted Least Squares (RULS) estimation method was conducted on FACTOR [31]. The Nonnormed Fit Index (NNFI), Comparative Fit Index (CFI), and Root Mean Square Error of Approximation (RMSEA) were employed to evaluate the goodness-of-fit. As recommended by Byrne [32], NNFI and CFI ≥ .95 along with RMSEA ≤ .08 would suggest good model fit.

Subsequently, IRT item parameters were estimated under the GRM model using the marginal maximum likelihood estimation. Seven parameters were estimated for each item, including one item-discrimination value (*a*) and six item-threshold values (*b*_*i*_), which are equal to the number of response options minus one and they represent an item’s sensitivity in differentiating among the various levels of the target trait. Based on the discrimination and threshold parameters, the item information functions (IIFs) describe the amount of information on the latent trait that a particular item provides. An item typically offers a larger amount of item information if it has a greater discriminating parameter (i.e., steeper slopes) and a broader range of threshold parameters along θ. Thus, to shorten the 21-item original CLS-H, we selected the items that offered higher information looking at the shape of each item information function, which clearly display the amount of item information along the latent trait.

To examine the precision of the shortened scale throughout the continuum of the latent trait (local reliability), the test information function (TIF) was compared with that of the original CLS-H scale. Specifically, the (I) can be transformed in *r* = 1-(1/I), which can be interpreted following the criteria proposed for the classical reliability indices [33]. Then, we computed the percent change in *r* to show that the scale precision did not drastically decrease moving from the original to the abbreviated version.

Finally, to compare the original and short forms, we estimated individual IRT scores based on the original and short forms and calculated the correlation between the two IRT scores as well as the average difference in the scores across individuals [19]. Then, using IRT person scores and measures of related psychological concepts, validity of both the original and shortened form of the CLS-H was investigated via Spearman’s rank correlation correlations. Bivariate correlations obtained for each version were compared using Lee and Preacher’s [34] test of the difference between two dependent correlation coefficients obtained from the same sample.

## Results

### Item selection for the shortened scale

#### Preliminary analyses

A parallel analysis based on minimum-rank FA as an extraction method was conducted which indicated support for a one-factor solution. Unrestricted factor analysis suggested that the one-factor model explained 45% of the variance and factor loadings ranged from .45 to .76. The one-factor model showed good fit indices (CFI = .98 [95% CI: .98–.99]; NNFI = .98 [95% CI: .98–.99]; RMSEA = .058 [95% CI: .053–.059].

#### Item parameters, measurement precision, and selection method

**Fig. 1:**
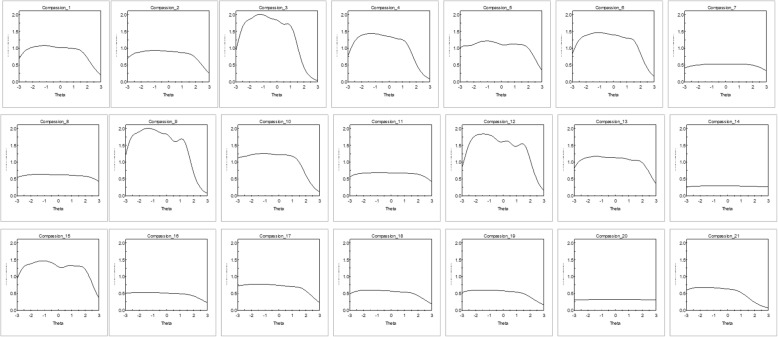
Item Information Function (IIF) of the 21 items of the Compassionate Love Scale for Humanity. *Legend*: Latent trait (Theta) is shown on the horizontal axis, and the amount of information yielded by the item at each trait level is shown on the vertical axis

**Fig. 2:**
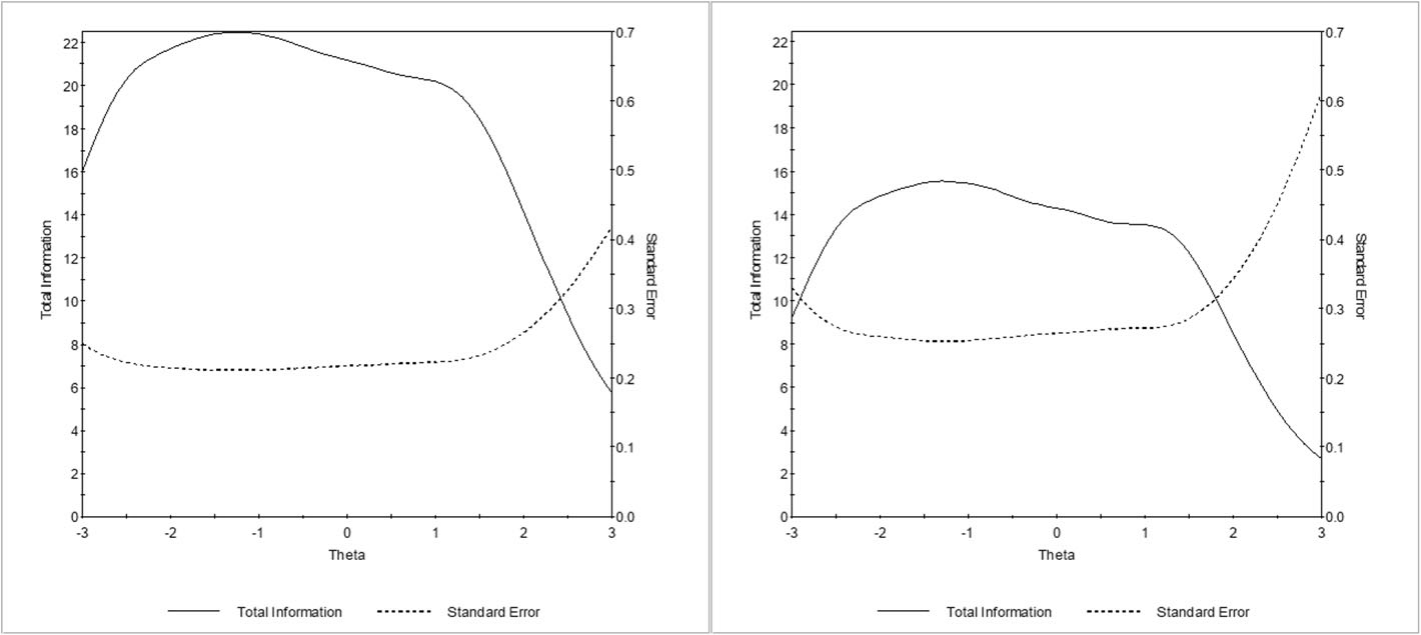
Test Information Function (TIF) of the original and short Compassionate Love Scale for Humanity. *Legend*: The original version is on the left and the short version is on the right. Latent trait (Theta) is shown on the horizontal axis, and the amount of information and the standard error yielded by the test at each trait level is shown on the vertical axis

In line with the factor analysis results, IRT analysis was performed under the unidimensional graded model. Item parameters are reported in Table 1 while the information carried out by each item is displayed graphically in Fig. 1. Item discrimination values ranged from 0.95 to 2.52. Category threshold values ranged from − 3.94 (lowest *b*_*1*_ value) to 3.40 (highest *b*_*6*_ value). Nonetheless, items that cover a wider range of the latent trait are the ones that convey lower information (item 7, 8, 14, and 20). Accordingly, the TIF (Fig. 2) shows that the scale information across the compassionate love continuum steadily drops starting from a theta level of about 1.50.

**Table 1:** Item response theory parameters of the full GRM for each item of the Compassionate Love for Humanity Scale (CLS-H)

*Items*	*Item Response Theory Parameters*						
	*a*	*b*_1_	*b*_2_	*b*_3_	*b*_4_	*b*_5_	*b*_6_
**1**	1.83	− 2.49	− 1.64	− 1.09	−0.52	0.50	1.53
**2**	1.71	−2.63	−1.65	−1.04	−0.36	0.57	1.70
**3**	**2.51**	**−2.40**	**− 1.62**	**− 1.23**	**− 0.77**	**0.00**	**1.00**
**4**	**2.12**	**−2.40**	**− 1.72**	**−1.23**	**−0.70**	**0.11**	**1.14**
**5**	**1.97**	**−2.92**	**−1.72**	**− 1.07**	**− 0.37**	**0.80**	**1.87**
**6**	**2.15**	**−2.45**	**−1.59**	**− 1.07**	**− 0.44**	**0.39**	**1.46**
**7**	1.29	−2.58	− 1.36	−0.53	0.45	1.33	2.20
**8**	1.41	−2.87	−1.82	− 1.11	−0.08	1.02	2.35
**9**	**2.52**	**−2.57**	**−1.78**	**−1.31**	**−0.74**	**0.08**	**1.22**
**10**	**2.01**	**−3.05**	**− 2.02**	**−1.26**	**−0.55**	**0.35**	**1.27**
**11**	1.47	−2.76	−1.71	−0.82	0.09	1.13	2.26
**12**	**2.41**	**−2.37**	**−1.78**	**− 1.25**	**− 0.68**	**0.34**	**1.53**
**13**	**1.94**	**−2.59**	**−1.67**	**−1.17**	**−0.24**	**0.71**	**1.89**
**14**	0.95	−3.27	−1.97	−0.75	0.38	1.79	3.57
**15**	**2.16**	**−2.54**	**−1.62**	**− 1.02**	**− 0.35**	**0.83**	**1.90**
**16**	1.28	−3.79	−2.63	−1.66	− 0.85	0.31	1.72
**17**	1.56	−3.36	−2.28	−1.51	− 0.79	0.26	1.62
**18**^a^	1.37	−2.71	−2.10	−1.18	−0.05	1.50	
**19**^a^	1.37	−2.93	−2.06	−1.06	−0.12	1.33	
**20**	1.01	−3.94	−1.99	−0.91	0.35	1.83	3.40
**21**^a^	1.46	−2.95	−2.22	−1.54	−0.61	0.67	

*Note: a* discrimination parameter, *b* threshold parameters; *GRM* graded response models. Bold indicates the item selected for the shortened scale using the information method. ^a^The first two response options were collapsed due to the low frequency of the first response category

Thus, we selected the items that conveyed higher information along the trait continuum (i.e., items 3, 4, 5, 6, 9, 10, 12, 13, and 15) aiming to maintain maximum precision and a trait coverage similar to the original form. Examining the content, we observed that the retained items addressed the key features of compassion as being aware of people’s adversity and discomfort (e.g. item 4), responding emotionally to their suffering (e.g. item 10), and desiring to act to alleviate people’s misery and distress (e.g. item 12), while we excluded items referring mainly to altruism (e.g., item 11), empathy (e.g., item 18), and kindness (e.g., item 21). The short CLS-H version (CLS-H-SF) consisting of nine items is reported in the Appendix.

### Psychometric characteristics of the CLS-H-SF (and comparison with the CLS-H)

#### Factor structure

As for the original version, parallel analysis indicated support for a one-factor solution of the shortened scale. The one-factor model showed good fit indices (CFI = .992 [95% CI: .990–.998]; NNFI = .990 [95% CI: .987–.995]; RMSEA = .054 [95% CI: .040–.061]. The single factor explained 59% of the variance and factor loadings ranged from .68 to .78.

#### Item parameters

Item discrimination values were very high ranging from 1.92 to 2.67. Category threshold values ranged from − 3.08 (lowest *b*_*1*_ value) to 1.92 (highest *b*_*6*_ value) indicating that the items of the shortened scale performed well in measuring a large spectrum of the underlying construct, but not for the higher levels.

#### Measurement precision

Each item conveyed a high amount of information. Subsequently, the TIF showed that the 9-item CLS-H is precise in measuring the different levels of the compassionate love construct continuum and it is similar to the TIF of the original scale in both shape and coverage across the compassionate love continuum (Fig. 2). Since the shortening of the scale inevitably results in the reduction of the amount of information provided by the scale, the drop was consistent with the large number of items that were removed (i.e., from 21 items to 9 items). However, it is important to note that the increase in standard errors never exceeds .10 standard errors along the theta range from − 2.8 to 2.4. To quantify the change in reliability, we computed the percentage change in reliability along the different trait levels (Table 2). Whereas it is less precise for the highest levels of the trait, these results confirm that the abbreviated scale maintains good accuracy from low to high levels of the trait.

**Table 2:** Reliability indices yielded by the original and short versions of the Compassionate Love for Humanity Scale (CLS-H and CLS-H-SF) for each level of the Theta latent trait

*Theta*	*Reliability*		
	*CLS-H*	*CLS-H-SF*	*% change*
*− 2.8*	0.94	0.91	4.21
*−2.4*	0.95	0.93	2.10
*−2*	0.95	0.93	2.10
*−1.6*	0.95	0.93	2.10
*−1.2*	0.95	0.94	1.05
*−0.8*	0.95	0.93	2.10
*−0.4*	0.95	0.93	2.10
*0*	0.95	0.93	2.10
*0.4*	0.95	0.93	2.10
*0.8*	0.95	0.93	2.10
*1.2*	0.95	0.92	3.16
*1.6*	0.94	0.92	2.13
*2.0*	0.93	0.91	2.15
*2.4*	0.90	0.88	2.22
*2.8*	0.86	0.70	18.60

#### Validity

First of all, IRT scores based on the original and 9-item short form were generated for each respondent. The correlation between these two scores was .95 and the mean difference in scores was −.0004. This difference was not significant (*t*(789) = − 04, *p* = .97), lending support for the correspondence between the two forms.

Bivariate correlations of both CLS-H and CLS-H-SF IRT scores with measures of related psychological concepts are reported in Table 3. As expected, compassionate love was positively associated with self-esteem, playfulness, positive affect, and life satisfaction and negatively associated with anxiety and negative affect. Calculation for the test of the differences between two dependent correlations showed that the investigated correlations were not statistically different when comparing the original scale and the shortened version, thus further providing validity evidence for the short-form (Table 3).

**Table 3:** Comparison of correlations between the original and short versions of the Compassionate Love for Humanity Scale (CLS-H and CLS-H-SF) IRT scores and all other variables in the study

	*CLS-H*	*CSL-H-SF*	*z*	*p value*
Self-Esteem	.145	.137	*0.70*	*.49*
Anxiety-State	−.122	−.122	*−0.00*	*1.00*
Playfulness	.347	.338	*0.83*	*.41*
Positive Affect	.119	.105	*1.21*	*.22*
Negative Affect	−.141	−.140	*0.09*	*.93*
Satisfaction with Life	.178	.161	*1.49*	*.069*

*Note*. *N* = 789. All correlations were significant at *p* < .01. The *z*-comparisons were between the correlation coefficients that were calculated using computer software provided by Lee & Preacher [33]

## Discussion

The present study aimed at developing a shortened version of the CLS-H that preserves the measurement integrity of the longer version and overcomes some content shortcomings of the original form that includes items more focused on empathy, kindness and altruism, which are constructs separate from compassionate love [1]. In particular, the present study was the first to investigate the item response theory parameters of the CLS-H and subsequently develop a shorter precise and valid brief version using about half of the items, resulting in the nine item CLS-H-SF. Using IRT information estimates, we selected the items that distinguished most adequately among individuals with different levels of compassionate love and fully covered the spectrum of the latent trait. At the same time, we paid attention to the item content and confirmed that items conveying higher information along the trait continuum were also more centered on the key features of compassionate love that reflected being aware of people’s adversity and discomfort, responding emotionally to their suffering, and desiring to act to alleviate people’s misery and distress [3]. Additionally, we observed that items that conveyed less information along the trait continuum were focused more on altruism, empathy, and kindness, which are related but distinct constructs [1].

The item selection allowed the obtainment of a brief, precise, and valid version of the CLS-H. Regardless of the inevitable shrinkages in reliability due to item elimination, the test information function showed that the obtained short form was adequately precise for a large range of the trait. Overall, values of reliability were consistently high across the continuum, whereas they decreased for the higher levels of the trait. As for validity testing, the short scale seems to maintain the same construct coverage of the original scale because we were able to replicate the previously observed pattern of correlations observed for the longer form [2, 35]. As expected, we confirmed the positive associations between compassionate love and measures of self-esteem, positive affect, and life satisfaction, as well as negative associations with negative affect and anxiety [2, 35].

The current study makes a useful and important contribution to research on compassionate love requiring a precise and concise measure. Employing IRT analyses techniques, we developed a precise and valid short form. This concise and more centered measure may provide added value in research and practice because it can be used to identify expressions of compassionate love across various contexts [3, 11], while highlighting the positive effects of compassionate love in promoting pro-social behaviors [1, 2, 4], engagement, and well-being [7–9]. The measure can also be used to implement interventions that seek to enhance people’s ability to give and receive compassion [12, 13]. In particular, a brief scale will allow researchers and practitioners to incorporate additional constructs into their survey to advance research in several ways. For example, the developed scale might contribute to the literature examining and expanding the nomological network of compassionate love, it can be employed in multivariate studies aiming at identifying the conditions that might foster and impede the expression of compassionate love, or used in research that employ a test-retest experimental design to understand how compassionate love might be fostered in individuals. The use of a shortened scale can add to the potential to increase our understanding of the relationships between compassionate love and other constructs as well as to develop intervention strategies to improve altruistic helping and prosocial behaviors. For example, the measure may be utilized in healthcare and education systems where compassionate love may impact clinical outcomes and quality of care [5, 6], patients’ well-being [7], and teachers engagement [9].

The current brief scale has several strengths when compared to the previously developed brief form of the CLS-H, the Santa Clara brief compassion scale (SCBCS) [17]. First of all, along with the general reliability, we were able to assess the local reliability (i.e., to display the precision of the CLS-H-SF along the different levels of the measured trait). Second, validity evidence was provided and, in particular, we assured that the shortened scale replicates the characteristics of the long version. Finally, the present study assessed the discrimination and location parameters of each item preserved for the short form of the measure. Thus, items that provided the most information along the latent continuum were preserved for the short form to maximize its effectiveness.

While this study provides a precise and valid measure of compassionate love, there are some limitations that highlight directions for future research. First, participants received the full version of the CLS-H, and therefore the potential impact of non-retained items on responding was not assessed. Thus, further investigation should employ the short scale to replicate and strengthen the current findings. Second, the uniform age of the sample might limit the generalizability of the current results. As such, future studies should be conducted with samples of different age distributions and other personal (e.g. gender) and demographic factors (e.g. education level, socio-economic indicators). Third, the range of validity measures was limited. Future studies should include the measurement of constructs that are expected to correlate positively with compassionate love, such as measures of prosocial behaviors [2, 14], but also negatively with factors associated with the Dark Tetrad (narcissism, Machiavellianism, psychopathy, sadism) [36]. Fourth, when compared to the original scale our shortened scale was less precise in measuring the high levels of compassionate love. Although this might be a minor issue, given that the loss in information was observed only for levels located over two standard deviations above the mean, it might pose a limitation in research aiming at assessing improvement in compassionate love. Therefore, future studies may consider introducing some changes in items to cover the higher end of the latent trait.

## Conclusion

Whereas some users may prefer the 21-item version of the CLS-H, we aimed to provide researchers and practitioners with a different scale option that they can choose based on their purposes/objectives (e.g., number of variables to be measured), study design (e.g., cross-sectional or longitudinal), and available resources (e.g., time). Using an IRT analytical approach and taking in account the item content, the current study suggests that the CLS-H-SF demonstrated strong reliability and validity in measuring compassionate love. Overall, the CLS-H-SF is a less time-consuming and effort-demanding tool that measures the construct well. As such, it can be used for future assessment protocols focused on compassionate love.

## Data Availability

The datasets used and/or analyzed during the current study are available from the corresponding author on reasonable request.

## Appendix

Items of the short Compassionate Love for Humanity Scale (CLS-H-SF).

**Table:**

1. When I hear about someone (a stranger) going through a difficult time, I feel a great deal of compassion for him or her. (3)	
2. It is easy for me to feel the pain (and joy) experienced by others, even though I do not know them. (4)	
3. If I encounter a stranger who needs help, I would do almost anything I could to help him or her. (5)	
4. I feel considerable compassionate love for people from everywhere. (6)	
5. I tend to feel compassion for people even though I do not know them. (9)	
6. One of the activities that provides me with the most meaning to my life is helping others in the world who need help. (10)	
7. I often have tender feelings toward people (strangers) when they seem to be in need. (12)	
8. I feel a selfless caring for most of mankind. (13)	
9. If a person (a stranger) is troubled, I usually feel extreme tenderness and caring. (15)	

*Note:* In brackets the item number of the original scale

## Abbreviations

CLS-H: Compassionate Love for Humanity Scale
CLS-H-SF: Compassionate Love for Humanity Scale Short Form
IIF: Item Information Function
IRT: Item Response Theory
SCBCS: Santa Clara Brief Compassion Scale
TIF: Test Information Function

## References

[1] Strauss C, Taylor BL, Gu J, Kuyken W, Baer R, Jones F (2016). What is compassion and how can we measure it? A review of definitions and measures. Clin Psychol Rev.

[2] Sprecher S, Fehr B (2005). Compassionate love for close others and humanity. J Soc Pers Relat.

[3] Kanov JM, Maitlis S, Worline MC, Dutton JE, Frost PJ, Lilius JM (2004). Compassion in organizational life. Am Behav Sci.

[4] Batson DC, Change J, Orr R, Rowland J (2002). Empathy, attitudes, and action: can feeling for a member of a stigmatized group motivate one to help the group?. Personal Soc Psychol Bull.

[5] Epstein RM, Franks P, Shields CG, Meldrum SC, Miller KN, Campbell TL, Fiscella K (2005). Patient-centered communication and diagnostic testing. Ann Fam Med.

[6] Najjar N, Davis LW, Beck-Coon K, Carney DC (2009). Compassion fatigue: a review of the research to date and relevance to cancer-care providers. J Health Psychol.

[7] Cosley BJ, McCoy SK, Saslow LR, Epel ES (2009). Is compassion for others stress buffering? Consequences of compassion and social support for physiological reactivity to stress. J Exp Soc Psychol.

[8] MacBeth A, Gumley A (2012). Exploring compassion: a meta-analysis of the association between self-compassion and psychopathology. Clin Psychol Rev.

[9] Eldor L, Shoshani A (2016). Caring relationships in school staff: exploring the link between compassion and teacher work engagement. Teach Teacher Edu.

[10] van Dierendonck D, Patterson K (2015). Compassionate love as a cornerstone of servant leadership: an integration of previous theorizing and research. J Bus Ethics.

[11] Brody S, Wright SC, Aron A, McLaughlin-Volpe T, Fehr B, Sprecher S, Underwood L (2009). Compassionate love for individuals in other social groups. The science of compassionate love: Theory, research, and applications.

[12] Saunders W (2013). Love + compassion = community. Growing compassionate love in Communities.

[13] Gilbert P (2010). The compassionate mind.

[14] Sprecher S, Fehr B, Zimmerman C (2007). Expectation for mood enhancement as a result of helping: the effects of gender and compassionate love. Sex Rol.

[15] Smith GT, McCarthy DM, Anderson KG (2000). On the sins of short-form development. Psychol Assess.

[16] Wanous JP, Reichers AE, Hudy MJ (1997). Overall job satisfaction: how good are single-item measures?. J Appl Psychol.

[17] Hwang JY, Plante T, Lackey K (2008). The development of the Santa Clara brief compassion scale: an abbreviation of Sprecher and Fehr’s compassionate love scale. Pastoral Psychol.

[18] Embreston SE, Reise SP (2000). Item response theory for psychologists.

[19] Edelen MO, Reeve BB (2007). Applying item response theory (IRT) modeling to questionnaire development, evaluation, and refinement. Qual Life Res.

[20] Hambleton RK, Swaminathan H, Rogers WH (1991). Fundamentals of item response theory.

[21] Proyer RT (2012). Development and initial assessment of a short measure for adult playfulness: the SMAP. Pers Indiv Diff.

[22] Rosenberg M (1965). Society and the adolescent self-image.

[23] Gray-Little B, Williams VS, Hancock TD (1997). An item response theory analysis of the Rosenberg self-esteem scale. Personal Soc Psychol Bull.

[24] Watson D, Clark LA, Tellegen A (1988). Development and validation of brief measures of positive and negative affect: the PANAS scales. J Pers Soc Psychol.

[25] Diener ED, Emmons RA, Larsen RJ, Griffin S (1985). The satisfaction with life scale. J Pers Ass.

[26] Lucas RE, Diener E, Suh E (1996). Discriminant validity of well-being measures. J Pers Soc Psychol.

[27] Cai L, Du Toit SHC, Thissen D (2011). IRTPRO: flexible, multidimensional, multiple categorical IRT modeling [computer software].

[28] Ostini R, Nering ML (2006). Polytomous item response theory models.

[29] Samejima F (1969). Estimation of latent ability using a response pattern of graded scores. Psychometrika.

[30] Samejima F, van der Linden WJ, Hambleton RK (1996). Graded response model. Handbook of modern item response theory.

[31] Lorenzo-Seva U, Ferrando PJ (2013). FACTOR 9.2: a comprehensive program for fitting exploratory and semi-confirmatory factor analysis and IRT models. Appl Psychol Meas.

[32] Byrne BM. A primer of LISREL: basic applications and programming for confirmatory factor analytic models. New York: Springer Science & Business Media; 2012.

[33] Thissen D, Wainer H (2000). Reliability and measurement precision. Computerized adaptive testing: a primer (2nd ed.).

[34] Lee IA, Preacher KJ (2013). Calculation for the test of the difference between two dependent correlations with one variable in common [software].

[35] Neff KD, Pommier E (2003). The relationship between self-compassion and other-focused concern among college undergraduates, community adults, and practicing meditators. Self Identity.

[36] Plouffe RA, Smith MM, Saklofske DH (2019). A psychometric investigation of the assessment of sadistic personality. Pers Indiv Differ.

